# Characterization of mouse models of synucleinopathies

**DOI:** 10.1002/alz70855_106153

**Published:** 2025-12-24

**Authors:** Latiyah TC Timothy, Vladislav Novikov, Kellly Summers, Thomas M. Durcan, Edward A Fon, Czarina Evangelista, Ravi S. Menon, Lisa M Saksida, Timothy J Bussey, Joel Watts, Vania F Prado, Marco AM Prado

**Affiliations:** ^1^ University of Western Ontario, London, ON, Canada; ^2^ Schulich School of Medicine and Dentistry, London, ON, Canada; ^3^ Robarts Research Institute, London, ON, Canada; ^4^ McGill University, Montreal, QC, Canada; ^5^ Montreal Neurological Institute, McGill University, Montreal, QC, Canada; ^6^ Centre for Functional and Metabolic Mapping, Robarts Research Institute, London, ON, Canada; ^7^ University of Toronto, Toronto, ON, Canada

## Abstract

**Background:**

Synucleinopathies, such as Parkinson's disease (PD) and dementia with Lewy bodies (DLB), are characterized by the aggregation of pathological alpha‐synuclein (αSyn). The M83 hemizygous (het) transgenic mouse model has been extensively used to study synucleinopathies following injection of human αSyn preformed fibrils (PFFs). However, like other transgenic mouse lines, M83 mice exhibit an aggressive phenotype. To explore alternative models, we attempted to replicate findings using wild‐type (WT) mice injected with mouse αSyn PFFs. Additionally, we generated novel knock‐in humanized αSyn mouse models expressing fully human wild‐type, PD‐associated (A53T), or DLB‐associated (E83Q) αSyn mutants.

**Method:**

M83 hemizygous mice injected with human PFFs were analyzed using light‐sheet microscopy. WT mice were injected with either mouse αSyn PFFs or PBS (control). Cognitive flexibility was assessed using the Two‐Choice Pairwise Visual Discrimination‐Reversal (PVD‐R) touchscreen task. Motor behavior was evaluated using Rotarod, CatWalk gait analysis, wire‐hang, grip force, and open field tests. Phosphorylated αSyn was detected via Western blotting and immunofluorescence. Humanized αSyn mouse models were generated in collaboration with Cyagen using CRISPR‐Cas9 and homologous recombination.

**Result:**

M83 mice exhibited early cognitive impairments and a disproportionately high accumulation of S129‐phosphorylated αSyn in the spinal cord, consistent with their severe motor deficits. Preliminary findings indicate no significant differences in cognitive performance between mouse PFF‐injected and control WT mice in the PVD‐R task. Furthermore, WT mice displayed little to no αSyn pathology, and only male WT PFF‐injected mice demonstrated mild motor deficits in Rotarod and wire‐hang tests approximately eight months post‐injection, despite minimal αSyn pathology. Western blot analysis confirmed that human αSyn is expressed in heterozygous humanized mouse models at levels comparable to endogenous mouse αSyn. Phosphorylated αSyn was also detected in the RIPA‐soluble fraction in mutant mice.

**Conclusion:**

Our findings highlight limitations in both the M83 and WT αSyn PFF models. Given these results, we will focus on humanized WT, A53T, and E83Q SNCA models, which are expected to provide more physiologically relevant αSyn expression and improved reliability for studying synucleinopathies. These models may enhance our understanding of disease progression and facilitate the identification of potential therapeutic targets.

